# The magnitude and extent of edge effects on vascular epiphytes across the Brazilian Atlantic Forest

**DOI:** 10.1038/s41598-020-75970-1

**Published:** 2020-11-02

**Authors:** Edicson Parra-Sanchez, Cristina Banks-Leite

**Affiliations:** Department of Life Sciences, Imperial College London, Silwood Park Campus, Ascot, SL5 7PY UK

**Keywords:** Tropical ecology, Plant ecology, Community ecology

## Abstract

Edge effects are ubiquitous landscape processes influencing over 70% of forest cover worldwide. However, little is known about how edge effects influence the vertical stratification of communities in forest fragments. We combined a spatially implicit and a spatially explicit approach to quantify the magnitude and extent of edge effects on canopy and understorey epiphytic plants in the Brazilian Atlantic Forest. Within the human-modified landscape, species richness, species abundance and community composition remained practically unchanged along the interior-edge gradient, pointing to severe biotic homogenisation at all strata. This is because the extent of edge effects reached at least 500 m, potentially leaving just 0.24% of the studied landscape unaffected by edges. We extrapolated our findings to the entire Atlantic Forest and found that just 19.4% of the total existing area is likely unaffected by edge effects and provide suitable habitat conditions for forest-dependent epiphytes. Our results suggest that the resources provided by the current forest cover might be insufficient to support the future of epiphyte communities. Preserving large continuous ‘intact’ forests is probably the only effective conservation strategy for vascular epiphytes.

## Introduction

The world’s old-growth forests currently account for only 18–24% of total forest cover^1,2^. In human-modified forests, habitat disturbance manifests itself through changes in habitat quality and area, connectivity, and importantly, edge effects^3^. Edges are the boundaries between two different habitats^4^, and edge effects can promote changes in abiotic conditions^5^ that disturb forest dynamics on either side of the edge, resulting in both positive and negative effects on biota^5–9^. Given that nearly 70% of the world’s remaining forests are within 1 km of an edge^3^, it is crucial that the magnitude and extent of edge effects are quantified for a wide range of taxa^10^ so that we can fully understand the impacts of anthropogenic disturbance on biodiversity and ecosystem functions.

The impacts of edge effects can be partitioned into two components—the magnitude (i.e. how different edge conditions are from interior habitats) and the extent (i.e. how far the influence reaches) of the edge influence^11,12^. Edge effects with large magnitudes have been reported in a wide variety of taxa and systems, from high rates of species turnover^13,14^ and increased tree mortality rates in the Amazon^15^, to lower species richness and abundance as well as shifts in community composition of beetles in temperate systems^16^. While the extent of edge effects on abiotic conditions only reaches up to 25–50 m into the forest^5^, forest-dependent species can be affected at much greater distances. For instance, mammals can be negatively affected up to 400 m into the forest^6^, while the effect on beetles can reach up to 1 km^10^, demonstrating that edge effects could drastically reduce the amount of suitable habitat for forest-dependent species.

One of the major consequences of edge effects is *biotic homogenisation*. This process involves the replacement of local, often endemic, biota with non-indigenous species or species with large geographic ranges^17,18^. For instance, studies have reported that rare and/or shade‐tolerant species are replaced by long‐lived, light‐demanding plant species^13^ and pioneer species become dominant throughout edge‐influenced habitats^14^. Therefore, biotic homogenisation can have profound effects on ecosystem function via the disruption of food web structure^19^, a reduction of functional richness^20^ or turnover in species traits^21^.

Various approaches for quantifying the extent and magnitude of edge effects have been proposed^22,23^. However, none have been reliably able to quantify edge effects in habitat fragments in which multiple edges interact. Forests fragments experience edge effects from all sides, and when the fragments are small, the effects of one edge may interact synergistically with those of an adjacent edge^7,10^. Lefebvre et al.^24^ developed a novel approach to quantify and map the magnitude and extent of edge effects on species abundances across landscapes. This approach incorporates a spatially explicit model to integrate the potential spill-over effects from different habitats, distance from nearby edges, and how these effects are modulated by habitat quality. This is the only approach available that explicitly accounts for the synergistic effects of multiple edges and allows species-specific responses to be identified^8^.

Identifying and understanding species-specific responses is important because while some species thrive in edge habitats^22,25^, others—such as species that are dependent on the forest interior—may become locally extinct^20^. Vascular epiphytes (i.e. vascular plants that establish a commensal relationship with their host^26^) are no exception. Previous studies have shown increasing species richness of epiphytes at edges^27^, while others have revealed a reduction in species richness and abundance near edges^28,29^. It is possible, however, that this controversy stems from where an epiphyte is found along the forest’s vertical axis—from canopy to understorey^30–32^. Canopy communities may be less affected by edges than the understorey because, even in intact forest, these species are more exposed to sunlight and wind and experience longer periods of drought than their understorey counterparts^33,34^, making them more resilient to the typical abiotic effects of edges between forested and cleared habitats. To our knowledge, no studies have investigated whether edge effects have distinct impacts along the vertical strata in Neotropical systems^35–37^.

Here we quantify the magnitude and extent of edge effects on understorey and canopy epiphyte communities in the Brazilian Atlantic Forest (BAF), a biodiversity hotspot dominated by small fragments of human-modified forest of which approximately 80% are smaller than 50 ha and are highly isolated from nearby fragments^3,38^. We specifically asked: what is the magnitude of edge effects on epiphytes, and does it differ between the canopy and the understorey? Also, what is the extent of the edge effects and what impacts do they have on the wider landscape? For the first question, we anticipated that epiphyte communities would be highly sensitive to the magnitude of the edge effects and that these effects would be stronger in the understorey than in the canopy stratum. For the second question, we expected edge effects to extend for hundreds of metres into the forest, resulting in strong impacts on the highly fragmented BAF. We sampled the matrix (i.e., pasture), forest edge and forest interior to quantify the magnitude of edge effects, as well as control sites far from the nearest edge to measure the extent of edge influence in human-modified forests.

## Results

We found 14,489 individual epiphytes from 201 species belonging to 18 families, including Orchidaceae (82 species) and Bromeliaceae (48 species) as the dominant families. The most abundant species were *Pleopeltis hirsutissima* (1,375 individuals) and *Octomeria gracilis* (1,080 individuals).

In the forest fragments, forest interiors harboured 51 species in total, of which 25% (13 species) were exclusive to this habitat (i.e. not found anywhere else). Forest edges hosted 29 species, of which 10% were exclusive (3 species), and the surrounding fragment matrix hosted 32 species, with 19% being exclusive (6 species). For the controls, continuous old-growth forest sites were home to 169 species total, of which 83% were exclusive to this habitat type (141 species), and pasture control sites contained 5 species with only one being exclusive. Of the 201 species observed, only seven were found across all habitats in old-growth forest, fragments and pastures.

### Magnitude of edge effects

**Figure 1:**
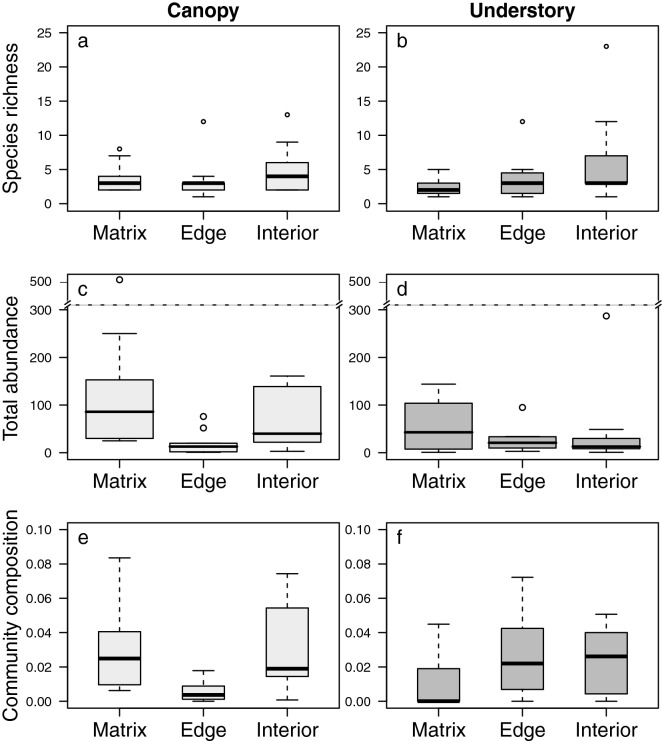
Magnitude of the edge influence on vascular epiphyte plants in forest fragments in the Brazilian Atlantic Forest. Results from generalised linear mixed-effects models (*n* = 12; alpha = 0.05) for the edge effects on species richness, total abundance of adult individuals, community composition along the canopy (**a**, **c** and **e**, respectively), and understorey stratum (**b**, **d** and **f**, respectively). Boxes show the median, 25th and 75th percentiles, error bars show 10th and 90th percentiles and points indicate the outliers. Dotted line in c and d represents axis break (310–450 individuals).

To assess the magnitude of edge effects for canopy and understorey communities, we used a paired design in which each of the 12 forest fragments had forest interior, forest edge and matrix plots (*n* = 36), each separated by 70–100 m. Overall, the magnitude of the edge effects on species richness was negligible across the interior-edge-matrix gradient in both strata—canopy and understorey—despite four forest edge plots being destitute of epiphytes in either stratum (GLMM, canopy, likelihood ratio test *X*^2^ = 4.622, *p* = 0.099; understorey, likelihood ratio test *X*^2^ = 0.489, *p* = 0.782; Fig. 1a–b, Supplementary Table S1). Similarly, we found no significant differences between forest interior and edge on total epiphyte abundance (pairwise comparisons with ‘glht’ function, Tukey method, canopy, *z* = – 1.806, *p* = 0.1680; understorey, *z* = – 0.754, *p* = 0.7312; Fig. 1c, Table S1). In contrast, we found a significantly larger number of individuals in the matrix than in the interior of both strata (canopy, *z* = 3.440, *p* = 0.0016; understorey, *z* = 2.523, *p* = 0.0312; Fig. 1d) than in the canopy edge (*z* = 5.209, *p* = 0.0001; Table S1).

On average, 80% of the species were found across the matrix-edge-interior gradient (Fig. 1e–f, Supplementary Figure S1, Tables S2–S3), which was reflected in the low magnitude of difference in community integrity between forest edge and interior on both strata (pairwise comparisons, canopy, *z* = 1.459, *p* = 0.3098; understorey, *z* = 1.804, *p* = 0.167; Table S2) and the matrix and forest edge across strata (canopy, *z* = 1.544, *p* = 0.269; understorey, *z* = 1.385, *p* = 0.347; Table S2). However, community integrity in the matrix, in both strata, were strongly different to forest interior (canopy, *z* = 3.515, *p* = 0.001; understorey, *z* = 2.956, *p* = 0.009; Table S2).

Edge effects have also homogenised forest structure, causing trees to present similar basal areas across the edge-interior gradient (estimate = − 15.46, *z* = − 2.144; Supplementary Table S4). As expected, matrix showed strong differences with forest interior and edge (matrix-interior, estimate = − 25.12, *z* = − 3.485; matrix-edge, estimate = − 9.667, *z* = 1.341; Table S4).

### Extent of edge effects

**Figure 2:**
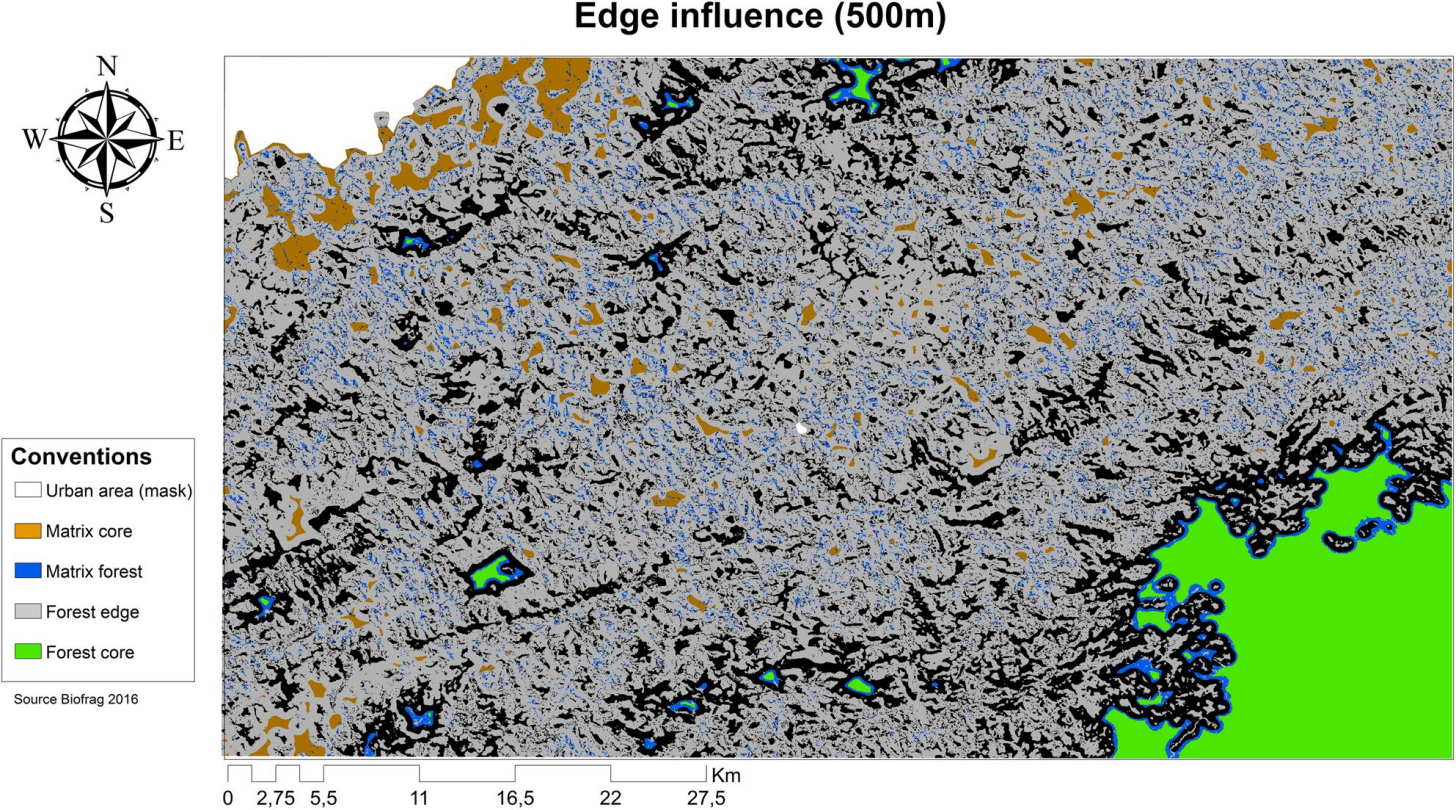
Map showing the landscape-level impacts of an edge influence (EI) of 500 m on epiphyte abundance. Colours depict the intensity of the EI on either forest or matrix. Green ‘forest core areas’ and orange ‘matrix core areas’ (i.e. areas far from a forest edge) experience no or very low EI (− 5–5 EI). Forest core areas can only be seen in the large protected areas in the southeast of the landscape and in a few large forest fragments. Blue represents forest areas that experience low EI (< − 5 EI). Black depicts the actual edges or areas where the interaction of multiple edges is present, consistent with the highest levels of EI (> 32 EI). Brown represents matrix areas experiencing medium to high positive influences from the edge (6–32 EI). Light grey shows urban areas (São Luiz do Paraitinga and Taubate, SP, Brazil). Map created by the authors using BIOFRAG (https://github.com/VeroL/BioFrag).

We quantified and mapped changes in species abundance across the landscape to estimate the extent of edge effects using a spatially explicit model, BIOFRAG®^24^. For this analysis, we included two additional habitat types far from any edge: old-growth forest in a national reserve (7,511–7,743 m from the nearest edge) as ‘control forest’ and trees within the matrix as ‘control pasture’ (491–702 m from the nearest edge; see Methods). We found that the extent of edge effects can reach at least 500 m (goodness-of-fit, rating: 0.97; Fig. 2), leaving approximately 18,100 ha (6.96%) of the studied landscape unaffected by edge effects. This includes 17,370 ha in the control forest (6.72% of the studied landscape), and just 725 ha outside the control forest (0.24% of the studied landscape). Epiphyte abundance in the control forest was practically unaffected by edge influence (mean = 1.4, SD +/− 0.02, *n* = 3), whereas a positive edge influence (i.e., forest influencing the matrix) was found in the matrix and control pasture epiphyte abundance (matrix mean = 26.9, SD + /− 6.3, *n* = 12; control pasture mean = 20.8, SD +/− 6.2, *n* = 3). Forest edge and forest interior epiphyte abundances were negatively affected by the edge (mean, edge mean = − 21.9, SD + /− 9.3, *n* = 12; interior: − 37.7, SD + /− 5.2, *n* = 12).

**Figure 3:**
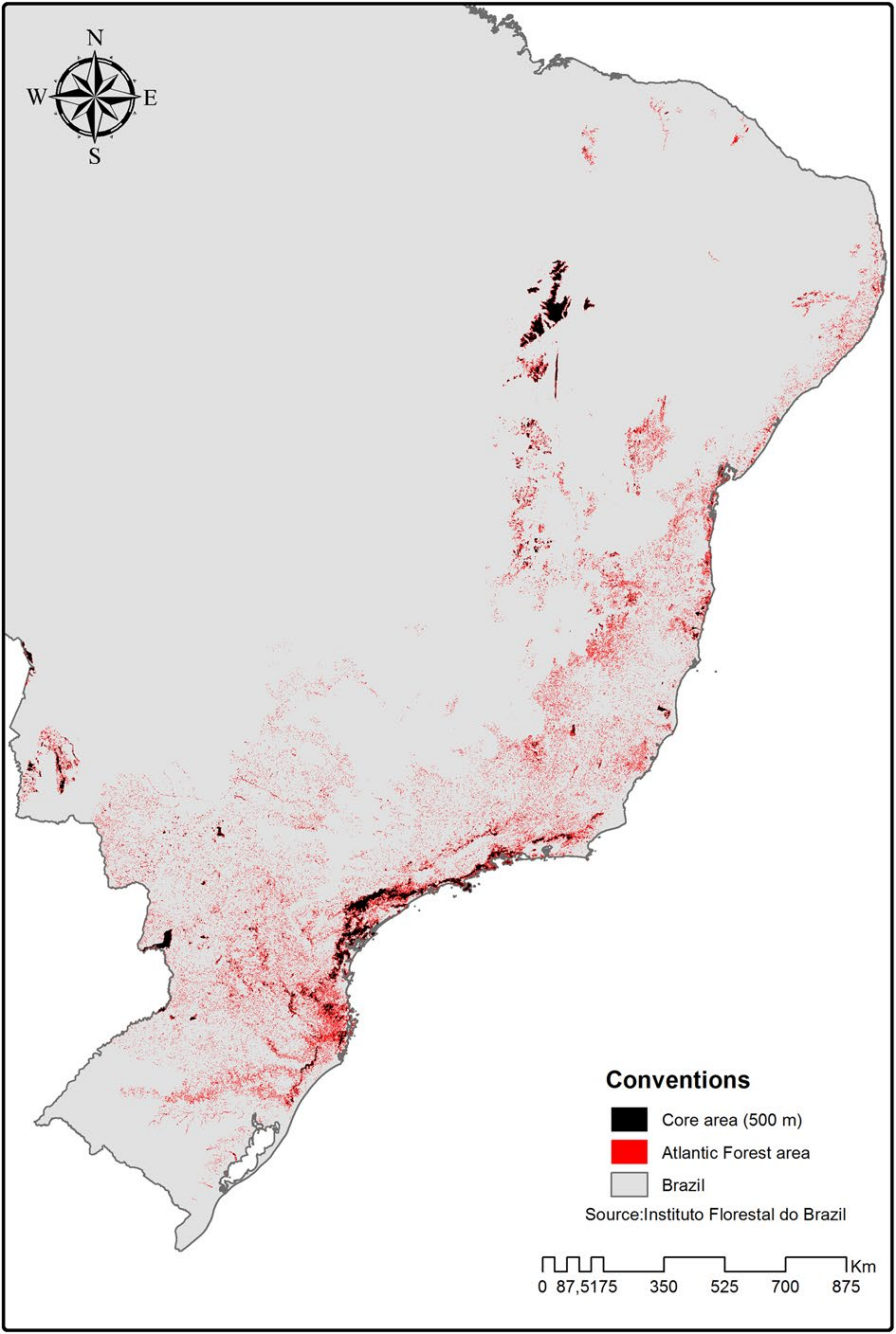
Amount of core area (in black) of forest fragments 500 m away from the edge in the Brazilian Atlantic Forest (BAF). Very few of the forest fragments in the BAF have a core area suitable for a high diversity of epiphytes. Map created by the authors using ArcGIS 10 (www.esri.com).

Subsequently, we extrapolated our landscape results to the entire BAF. Currently, the BAF has only 17.5 Mha of forest (roughly 12% of its original extent of 143 Mha^38^, Supplementary Table S5) which consists of remnants and regrowth forests. If we consider an edge extent of 500 m across the entire realm, then just 3.3 Mha or 19.4% of this forested area can be considered core area, or ‘free of edge effects’. Much of this core area is found only within large protected areas. Of the roughly 265,000 forest fragments in the BAF, only 1.7% have a core area unaffected by edge effects at the 500 m extent. Moreover, just 0.3% of these fragments (806 fragments) have a core area larger than 150 ha (Fig. 3).

Finally, we used the BIOFRAG®^24^ model to estimate species’ habitat preferences. In the canopy stratum, species that rely on forest interior conditions (‘forest core species’) were the most representative group (83%, 149 species), followed by forest edge (6.7%, 11 species) and matrix edge species (1.8%, 3 species). In contrast, the understorey stratum was dominated by forest edge species (50%, 36 species), followed by forest core species (32%, 25 species) and matrix edge species (12.7%, 9 species).

## Discussion

Our results provide evidence that (i) canopy and understorey strata respond similarly to edge effects, (ii) the magnitude of edge effects within fragments is low in both strata and (iii) edge effects extend far into forest interiors (> 500 m), leaving only 0.24% of the studied human-modified landscape ‘free of edge effects’. Together, these results suggest that there is a high degree of biotic homogenisation across the human-modified landscape and that the majority of the BAF is under this influence, as it has only 3.3 Mha (19.4% of the total BAF) of habitat further than 500 m from an edge. Our results strongly suggest that the future of vascular epiphytes in the BAF depends entirely on the protection of large continuous areas of pristine forest.

We found that species richness, total abundance, community integrity and tree structure did not differ across the interior-edge gradient. Counterintuitively, this suggests that forest fragments are under a high impact of edge effects, which extend all the way into forest interiors. This apparent low magnitude of edge effects within fragments is the result of a dramatic level of biotic homogenisation across the human-modified landscape. For instance, six species that occurred from the matrix to the interior were all widespread disturbance-tolerant species (*Aechmea vanhoutteana*, *Pleopeltis hirsutissima*, *Serpocaulon catharinae*, *S. latipes*, *Tillandsia gardneri*, and *T. geminiflora*). These species also represent 50% of the total species pool of the matrix, 28% of the edge and 17% of the interior, indicating the potential non-random species turnover from forest specialists to disturbance-tolerant species. Furthermore, the similar forest structure between edge and interior might have cascading effects on epiphyte communities by reducing habitat heterogeneity and habitat availability as a result of reduced tree growth^39^ and lower carbon stocks^40^.

Using the approach developed by Lefebvre et al.^24^, we were able to estimate that edge influences extend at least 500 m into the forest interior. These results add to the increasing evidence that edge effects might penetrate forest fragments as far as 1 km^6,23^. Furthermore, these results highlight the advantages of this model for measuring the extent of edge effects in human-modified landscapes as they allow researchers to better understand the synergies in edge effects when multiple edges are in close contact. It is important to mention, however, that although we estimated a 500 m extent of edge influence, we cannot exclude the possibility that the effects may extend even further within human-modified forests. The high degree of fragmentation in our study landscape hampered our ability to explore larger forest fragments containing areas farther than 500 m from an edge. Still, our results show that vascular epiphytes are more sensitive than the majority of vertebrates, which are influenced by edge effects as far as 200–400 m from an edge^6^. Only 31% of mammals studied worldwide depend on ‘forest core’ conditions^6^. However, we found that 83% of epiphyte species, including 10 species of conservation concern (VU and EX; Supplementary Table S6), were classified as ‘forest core’ species, surviving only in old-growth forest.

The future of epiphytes in the BAF is quite uncertain. Only 19.4% of the current forest (3.3 Mha) provides habitat for the forest core epiphytes. Moreover, only 0.3% of the roughly 265,000 fragments in the BAF have core areas larger than 150 ha. The extent of edge effects leaves vascular epiphytes with a much-reduced amount of ‘effective’ habitat area in the landscape^6,8,41^. Furthermore, intrinsic biological constraints of epiphytes—such as low seed survival^42^, pronounced slow growth rate^43^, higher mortality rate than tropical trees^44^, and the absence of seed banks for later re-colonization^43^—reduce the likelihood of long-term survival of forest-dependent species inside forest fragments. Therefore, the resources provided by the current forest fragments and the wider landscape itself might be insufficient to support abundant forest core species in the future.

Extrapolating landscape-level findings across the entire BAF region should be interpreted with caution. The extent of edge effects may vary between forest types (i.e. montane, lowland, semi-deciduous and ombrophilous forest) or forest successional stages (i.e. early, intermediate, and advanced stages), leading to certain forest fragments in the BAF being more (or less) impacted by edge effects than our model suggests. However, we could not incorporate these sources of variation into our model at this stage for two reasons. First, edge effects have not yet been quantified across different forest types, and second, differentiation between successional stages using satellite imagery has proven difficult thus far^45^, especially across large spatial scales^46,47^. Also, tree communities in forest edges can become similar in structure and biomass to early successional stages^13,14,48^, making it difficult to differentiate between forest edges and interiors^40^. Thus, it would be nearly impossible to distinguish the effects of successional stage from edge effects. Future work that attempts to unravel spatial variability in edge effects across different forest types and successional stages is crucial. Nevertheless, our extrapolation exercise builds upon the protocols of previous studies^3,6,49^. Additionally, our estimation of 500 m is aligned with the extent of 400 m reported for threatened mammals in the BAF^6^ and provides novel insights into the potential influences of edge effects at a regional scale.

Our study offers a holistic view of the ability of human-modified forests to sustain biodiversity when edge effects are quantified across the vertical and horizontal gradients in a human-modified landscape. The conservation value of disturbed and secondary forests has been shown to be high for trees^50^ birds, mammals, amphibians^49^ and invertebrates^51^, as well as for the provision of ecosystem functions^50^. However, this value may not extend to epiphytes in highly modified landscapes. The general decline in abundance of forest core epiphyte species and the invasion of matrix species, together with the well-documented slow recovery time of epiphyte communities after human disturbance^52,53^, make epiphytes one of the most sensitive groups to habitat loss studied to date. The dramatic impact of edge effects on epiphyte species and communities might translate into a low provision of ecological functions of these human-modified forests. Epiphytes benefit several other taxonomic groups. Their experimental removal drives a decline in species richness in birds^54^, invertebrates^55^, and herpetofauna^56^. Other functions would also be compromised after extirpation of vascular epiphytes, such as carbon sequestration via biomass production, water regulation, and modulation in light intensity along the vertical gradient^43,57,58^.

Ideally, conservation strategies to maintain vascular epiphytes in the BAF should prioritise the protection and enlargement of core forest areas. Restoration actions that reduce the perimeter-area ratio of fragments or expand the narrowest sections of large fragments could also increase the extent of core habitat; however, there is no guarantee that epiphytes would benefit, as many are highly dependent on old-growth forest conditions^59^. Nonetheless, as only a very small proportion of the forest fragments in the BAF have a core area, it is crucial that conditions are improved if we aim to preserve species in the long term. Meanwhile, protecting large continuous old-growth forests currently seems the only pragmatic strategy to maintain vascular epiphytes in the biodiversity hotspot of the Atlantic Forest.

## Methods

### Study area

**Figure 4:**
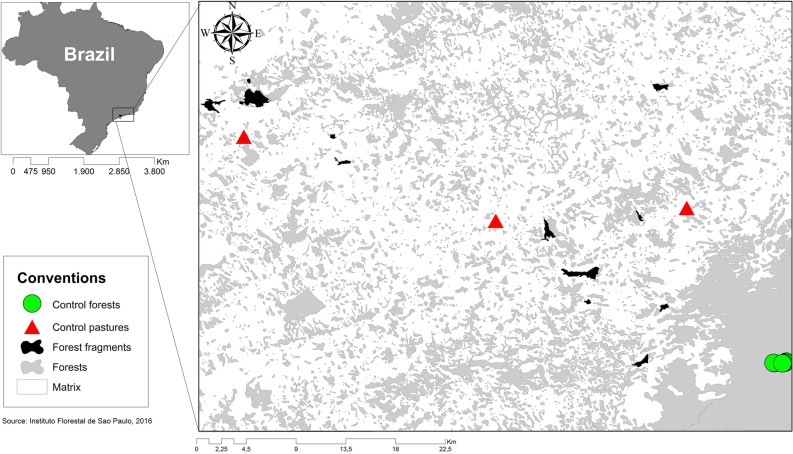
Map of the study area, showing the locations of fragments and sampling points within the Brazilian Atlantic Forest. Study design: grey areas represent forest fragments, white the matrix, black the sampled forests, triangles the control forests, and diamonds the control matrix. Map created by the authors using ArcGIS 10 (www.esri.com).

The study area is located in the BAF in the state of São Paulo (Fig. 4). The landscape studied has 28% forest cover in various successional stages with an average forest fragment size of 15 ha embedded within a non-forest matrix dominated by pasture. Study sites were a part of the ‘Biodiversity and Ecosystem Functioning in Degraded and Recovering Amazonian and Atlantic Forests’ (ECOFOR) research project (forest selection protocol in supplementary material). Fieldwork was conducted between May 2015 and July 2016.

**Figure 5:**
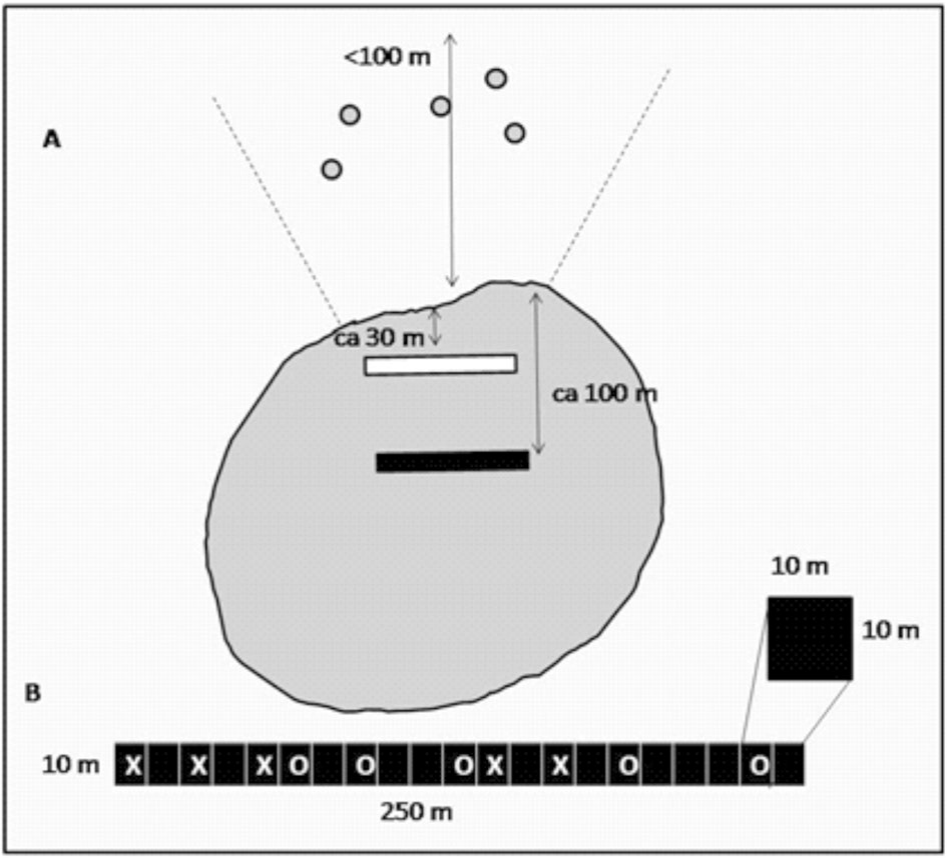
Sampling design for forest fragments. A) Black rectangle represents the forest fragment interior plot, white rectangle the forest fragment edge plot, arrows the distance from the edge fragment, and grey circles denote isolated tree plots in the matrix habitat. B) 10 × 250 m plot subdivided into 25 subplots of 10 × 10 m; letters represent an example of the sampling at the understorey strata, ‘X’ for canopy sampling and ‘O’ for random sampling.

Our sampling design consisted of 12 forest fragments and 6 control sites. Forest fragments were surrounded by pastures with isolated trees. In each fragment, we sampled the forest interior, the forest edge and the adjacent matrix (Fig. 5). Forest fragment interior plots were surveyed at 100 m from the nearest edge, forest edge plots were 30 m away from the edge, and matrix plots were located around isolated trees up to 100 m away from, and parallel to, the fragment edge. Trees were 10–50 m away from each other. In the forest interior and forest edge, we sampled a plot of 10 × 250 m subdivided into 25 subplots of 10 × 10 m. In the matrix, we sampled plots at 80–100 m away from the forest edge and parallel to the forest fragment, preserving the same sample area and design as the other plots (10 × 250 m).

We used two types of control sites: three plots of control pasture and three plots of control forest. Control pasture consisted of open matrix sites dominated by species of Poaceae with sparse trees, and the control forest consisted of an old-growth continuous forest in the Santa Virginia Nucleus of Serra do Mar State Park. In the control pasture, plots were placed 491–702 m away from the nearest discrete edge. We sampled plots of 10 × 250 m subdivided into 25 subplots of 10 × 10 m. In the control forest, we surveyed 100 × 100 m plots that had been established during a previous study^60^ as we were unable to open new trails and/or disturb the vegetation. Hence, we made a small modification to our approach to preserve the same sampling area per plot (2500 m^2^) and distance between trees (10–50 m) as in the forest fragments. We set up our sampling plot along the 100 m wide side of the original plot, then subdivided it into subplots of 10 × 10 m, as done in the fragments, for a total area of 1000 m^2^. We then repeated this to add another 1000 m^2^, and finally, another five subplots were added, accounting for 500 m^2^ for a total of 2500 m^2^. Control forest plots were 381–1150 m from each other, and we did not detect spatial autocorrelation in species composition (Mantel test, canopy, *p* = 0.168; understorey, *p* = 0.333).

### Epiphyte sampling

Epiphytes were sampled in the canopy and understorey strata. The canopy was sampled throughout Johansson’s tree sections III to V^61^ using a single rope technique. Branches were reached manually up to 5 m from the main trunk, while outer branches were inspected by binoculars and, when possible, surveyed using a pole. In the understorey, epiphytes up to 2 m high were recorded on trees with < 10 cm diameter at breast height (DBH). In each plot, we sampled the canopy of five trees; we randomly selected an additional five 10 × 10 m subplots for understorey sampling (Fig. 5).

Vascular epiphytes were surveyed in the canopy of 270 trees and in the understorey of 3,127 trees in 42 sampling plots (12 matrix, 12 forest edge, 12 forest interior, 3 control pastures, and 3 control forest). For both the canopy and the understorey plots, we recorded the number of epiphyte species and individuals on each tree. Following Sanford^62^ we defined an individual as a set of singular stems spatially separated from another set of stems of the same species. All individuals in early ontogeny stages and morphospecies were excluded. Species determination was conducted following specialised literature and consultation of experts. Nomenclatural standardisation was based on ‘The Plant List’ database names (The Plant List, 2010, using the r package ‘Taxonstand’^63^).

We also evaluated whether forest structure differed across habitat types (forest interior-edge-matrix). The average basal area per plot was used as a proxy for forest structure, and it was measured using all trees within the plot with > 10 cm DBH.

### Response variables

We measured observed species richness and total abundance at the plot scale. We used observed richness as it does not differ significantly from other rarefaction metrics (such as Hill numbers *q* = 0; see extended explanation in supplementary material) and it is more easily interpretable, particularly by practitioners and landscape managers. Changes in community integrity were measured as the difference in composition between each sampled plot in the human modified landscape and control forests. Hence, community integrity is an extension of beta diversity between two targeted habitat types^49,64,65^. Community composition was quantified using Bray–Curtis dissimilarity index with abundance data. Additionally, we used the permutation test for homogeneity of multivariate dispersion^66^ at both strata to assess the multivariate homogeneity of variance in epiphyte community composition across habitat types (interior-edge-matrix).

### Magnitude of edge effects

We fitted generalised linear mixed effect models (GLMMs) in which species richness and abundance were modelled with a negative binomial distribution using maximum likelihood estimation via Template Model Builder (TMB). Community integrity was fitted with Gaussian distribution in zero-inflated linear mixed effect models (LMMs) to account for the zero-inflated nature of the data (9 and 19 entries with a value of 0 in community integrity in the canopy and understorey, respectively). We also estimated the effect of habitat type on tree basal area with a Gaussian distribution error LMM. As fixed effects, we used the three habitat types: forest interior, edge, and matrix without interactions (alpha: 0.05, two-sided test). We included site as a random intercept to account for the nested structure of our sampling design (*n* = 18). Likelihood ratio tests were used to determine model significance by comparing models with habitat type to a null model with no predictor. Additionally, sampling effort (i.e. averaged tree basal area per plot) was included as an offset parameter in species richness and total abundance models. We tested pairwise effects among plots in all models using simultaneous linear hypothesis testing and Tukey test with Bonferroni-Holm correction (R package ‘multcomp’^67^). Visual inspection of residual plots did not reveal any clear deviations from homoscedasticity, normality or spatial autocorrelation (‘testResiduals’ in the Dharma r package^68^). Additionally, we tested for overdispersion (as the generalised Pearson *X*^2^ statistic divided by the number of observations), diagnosis of the variance–covariance matrix and the inference of the fixed effects estimated as the squared correlation between the response and the predicted value (functions ‘overdisp_fun’, ‘diagnose_vcov’, ‘cor’ and ‘cor’, respectively, from https://cran.r-project.org/web/packages/glmmTMB/vignettes/troubleshooting.html).

### Extent of edge effects

We mapped and quantified changes in the abundance of epiphyte species at the landscape scale using the Lefebvre et al.^24^ approach, which allowed us to characterize edge response and habitat preference per species based on their abundance. Here, we briefly summarise the method, but the full description can be found in Pfeifer et al.^6^ and at https://github.com/verol. This approach defines two spatially explicit metrics. The first is edge influence, which assesses the configuration of the landscape and calculates the local variation in percentage of tree cover within a 1 km radius. This metric ranges from 0 (in edge-free landscapes) to 100 (pixels surrounded by a different habitat). The value of edge influence can be positive (where matrix habitats are under the influence of the forest) or negative (where forest habitats are under the influence of the matrix), and values around zero correspond to either matrix core or forest core, far from any edge. In practice, edge influence characterises the extent of edge effects on both sides of the edge, and the sign determines the direction of the effect. The second metric is edge sensitivity of species, and this is a measure of preference for a certain habitat type. This metric ranges from 0 (non-edge sensitive species) to ± 1.0 (species exclusive to a particular habitat away from the edge) and the sign determines the direction of the preference (negative towards the matrix, positive towards the forest, and zero towards either matrix core or forest core).

To assess edge influence and species sensitivity, we used the species’ abundance matrices and a matrix with the coordinates of our sampling plots. The land cover maps were based on Hansen et al.^69^ map, which defines tree cover as canopy closure for all vegetation taller than 5 m; each pixel has a value between 0 and 100%. We later complemented this map with MapBiomas^70^ to identify tree cover categories not identifiable from Hansen’s map, i.e. tree plantations. We also excluded urban areas.

Additionally, the naive Bayesian classifier included in BIOFRAG aims to categorise species responses to edges. Species were categorised according to their abundance along the gradient as i) forest-core (i.e. highest abundance in the forest interiors), ii) forest-edge (i.e. highest abundance in the forest edge), iii) matrix-core (i.e. highest abundance in the matrix interior), and iv) matrix-edge (i.e. highest abundance in the matrix edge).

We performed the extent of edge influence analysis at five distances (250 m, 500 m, 750 m, 1000 m, and 2000 m) and selected the model with the highest rating based on the estimation of ‘how well the spatial distribution of the census points enables us to assess the species’ edge response’^24^. BIOFRAG’s rating is a measure of ‘goodness-of-fit’ of the output against the data; the closer it is to 1, the better the predictive model. Control plots allow for calibration of the species responses to edge influences and therefore the extent of the edge influence area. We calculated forest area in ArcGIS software^71^. Finally, we extrapolated our edge influence predictions across the entire 143 million hectares covered by the BAF. We used the map of ‘SOS Mata Atlantica’^72^. We calculated the buffer area within forest fragments with discrete borders in ArcGIS.

Statistical analyses were conducted in R^73^ with the packages ‘vegan’ (version 2.2–1^74^), ‘glmmTMB’^75^, ‘lme4’^76^, ‘MuMIn’^77^, and ‘multcomp’^67^. The extent of edge effects was calculated with BIOFRAG^24^.

### Ethic statement

Fieldwork in the human-modified forests was carried out on private properties with each landowner’s permission. Sampling in the control forest was done under the permission COTEC: 260108 – 002.959/2016.

## Supplementary information

Supplementary Information 1.

Supplementary Information 2.

## Data Availability

The datasets generated and analysed during the current study are available along with the R codes on Dataset 1 to ensure replicability.
