# Supplementary material for: The magnitude and extent of edge effects on vascular epiphytes across the Brazilian Atlantic Forest
Source: Sci Rep. 2020 Nov 2;10:18847. doi: 10.1038/s41598-020-75970-1 (PMC7606527; doi:10.1038/s41598-020-75970-1)
Supplement: Supplementary file 1 — Supplementary Information 1. [file 41598_2020_75970_MOESM1_ESM.docx]

## Supplementary material

**The magnitude and extent of edge effects on vascular epiphytes across the Brazilian Atlantic Forest**

Edicson PARRA-SANCHEZ^*^

Cristina BANKS-LEITE.

**ECOFOR sampling design**

Our 12 studied forest fragments were selected based upon ECOFOR protocols. The ECOFOR team selected focal fragments based on forest area and connectivity, using the distribution and range of values as well as practical considerations, such as the minimum size needed to establish plots, ability to be assessed from the road, presence of discrete edges, and embedding in a matrix of pastureland.

Native forest map was created using the forest cover map obtained from Instituto Florestal do Estado de São Paulo^1^. The map is classified into forest and non-forest fragments with a resolution of 30 m and projected in SAD 1969 UTM Zone 23S. Analyses were done in Fragstats^2^ and ArcGIS 10.1 ArcMap software (ESRI, CA, USA).

Forest fragments were categorised into small (< 50 ha) and large (> 50 ha) fragments and into different levels of connectivity. Connectivity was based on the proximity index^3^ (within 800 m) for all fragments in the study area. The proximity index accounts for the surrounding forest fragments (within the defined radius of 800 m) and weights the distance from the focal forest fragment to each nearby fragment^3^. The proximity index was log10 transformed and categorised into connected (0–3) or not connected (> 3). Selected forests were then filtered out based on fragment shape. The shape metric was assessed to ensure the existence of enough core area to establish sampling plots 100 m from the edge. Values of shape < 2 were chosen for small fragments and < 4 for large fragments. These values were selected based on core area calculations and visual comparisons.

**Hill numbers**

Hill numbers quantify the species diversity of a community relative to their abundances^4,5^ and are believed to more closely align with the effective number of species. Hill numbers include the three most widely used species diversity measures as special cases: species richness (*q* = 0), Shannon diversity (*q* = 1) and Simpson diversity (*q* = 2). We contrasted our measures of observed richness to Hill numbers of order *q* = 0 obtained from R package ‘iNEX’ with abundance data^6^ via a ‘Pearson correlation’. Empirical data was, unsurprisingly, highly correlated with Hill numbers across all habitat types (t-test, canopy, correlation = 0.997, *p* < 0.001; understory, correlation = 0.850, *p* < 0.001). We report observed species data as we believe practitioners and landscape managers are more familiar with species richness than with Hill numbers.

**Spatial autocorrelation within control forest**

Spatial autocorrelation of community composition in the control forest was assessed with a Mantel test. Significance was tested with the Monte-Carlo permutation test (*n* = 999) of the R function ‘mantel.randtest’ of the package ‘ade4’^7^.

Table S1. Magnitude of the edge effects on species richness and total abundance of vascular epiphytes across strata in human-modified forests in the Brazilian Atlantic Forest. Models were fitted with negative binomial distribution, and multiple linear comparisons with post-hoc Tukey method and Bonferroni-Hom correction. We used averaged tree basal area per plot as an offset parameter, and implemented a likelihood test of the models against a null model (intercept = 1). Table presents estimates (Estimates), standard error (Std. Error), *z*-statistic and its respective *p*-value (Pr (>|*z*|)) of the fixed effects; and model inference, and the likelihood ratio test *chi-*square statistic (*X*^2^) and respective *p*-value (Pr(>*X*^2^)). In bold statistically significant *p*-values (alpha = 0.05).

|  |  | | | | | | **Model inference** | | |
| --- | --- | --- | --- | --- | --- | --- | --- | --- | --- |
|  |  | **Habitat type** | **Estimate** | **Std-Error** | **z-value** | **Pr (>\|z\|)** | **Inference** | ***X*^2^** | **Pr(>*X*^2^)** |
| **Canopy** | **Richness** | Interior-Edge | -0.103 | 0.480 | -0.215 | 0.975 | 0.050 | 4.49 | 0.106 |
|  |  | Matrix-Interior | 0.811 | 0.459 | 1.766 | 0.181 |  | | |
|  |  | Matrix-Edge | 0.914 | 0.477 | 1.918 | 0.133 |  |  |  |
|  | **Abundance** | Interior-Edge | -1.065 | 0.590 | -1.806 | 0.168 | 0.106 | 19.54 | **<0.001** |
|  |  | Matrix-Interior | 2.003 | 0.582 | 3.440 | **0.002** |  | | |
|  |  | Matrix-Edge | 3.069 | 0.589 | 5.209 | **<0.001** |  |  |  |
| **Understory** | **Richness** | Interior-Edge | -0.339 | 0.492 | -0.688 | 0.770 | 0.084 | 7.42 | 0.783 |
|  |  | Matrix-Interior | -0.090 | 0.513 | -0.175 | 0.983 |  | | |
|  |  | Matrix-Edge | 0.249 | 0.542 | 0.460 | 0.890 |  |  |  |
|  | **Abundance** | Interior-Edge | -0.540 | 0.717 | -0.754 | 0.731 | 0.017 | 6.41 | **0.040** |
|  |  | Matrix-Interior | 1.269 | 0.714 | 1.778 | 0.177 |  | | |
|  |  | Matrix-Edge | 1.809 | 0.717 | 2.523 | **0.031** |  |  |  |

**Table S2**. Magnitude of the edge effects on community integrity of vascular epiphytes across strat**a** in human-modified forests in the Brazilian Atlantic Forest. A multiple comparison zero-inflated independent linear mixed model was fitted with Gaussian distribution. We used averaged tree basal area per plot as an offset parameter, and implemented a likelihood test of the models against a null model (intercept = 1). Table presents the estimates (Estimates), standard error (Std. Error), *z*-statistics and its re**s**pective *p*-value (Pr (>|*z*|)) of the fixed effects; alongside to model inference, and the likelihood ratio test chi square statistic (*X*^2^) and respective *p***-**value (Pr(>*X^2^*)). In bold statistically significant *z*-statistics (alpha =0.05).

| **Model inference** | | |  |  |  |  |  |  |
| --- | --- | --- | --- | --- | --- | --- | --- | --- |
|  | **Habitat type** | **Estimate** | **Std. Error** | ***z-*value** | **Pr (>\|*z*\|)** | **Inference** | ***X*^2^** | **Pr(>*X*^2^)** |
| **Canopy** | Interior-Edge | 0.563 | 0.386 | 1.459 | 0.310 | 0.0445 | 9.116 | **0.010** |
|  | Matrix-Interior | 1.159 | 0.330 | 3.515 | **0.001** |  | | |
|  | Matrix-Edge | 0.596 | 0.386 | 1.544 | 0.269 |  |  |  |
| **Understory** | Interior-Edge | 0.709 | 0.393 | 1.804 | 0.167 | 0.0108 | 6.957 | **0.030** |
|  | Matrix-Interior | 1.383 | 0.468 | 2.956 | **0.009** |  | | |
|  | Matrix-Edge | 0.675 | 0.487 | 1.385 | 0.347 |  |  |  |

**Table S3.** Total and relative proportion of exclusive and shared species of vascular epiphytes across canopy and understory stratum in the control forest, forest interior, forest edge and matrix in the studied area in the Brazilian Atlantic Forest.

| **All strata** | **Total** | **Exclusive** | **Shared** | **% exclusive** | **% shared** |
| --- | --- | --- | --- | --- | --- |
| **Control forest** | 169 | 141 | 28 | 83 | 17 |
| **Forest interior** | 51 | 13 | 38 | 25 | 75 |
| **Forest edge** | 29 | 3 | 26 | 10 | 90 |
| **Matrix** | 32 | 6 | 26 | 19 | 81 |
| **Canopy** | **Total** | **Exclusive** | **Shared** | **% exclusive** | **% shared** |
| **Control forest** | 168 | 140 | 28 | 83.3 | 16.7 |
| **Forest interior** | 23 | 5 | 17 | 26.1 | 73.9 |
| **Forest edge** | 16 | 1 | 15 | 6.3 | 93.8 |
| **Matrix** | 27 | 3 | 24 | 11.1 | 88.9 |
| **Understory** | **Total** | **Exclusive** | **Shared** | **% exclusive** | **% shared** |
| **Control forest** | 44 | 21 | 23 | 47.8 | 52.2 |
| **Forest interior** | 40 | 8 | 32 | 20 | 80 |
| **Forest edge** | 19 | 2 | 17 | 10.5 | 89.5 |
| **Matrix** | 9 | 3 | 6 | 32.3 | 66.7 |

**Table S4.** Magnitude of the edge effects on forest structure in human-modified forests in the Brazilian Atlantic Forest. Tree basal area pairwise comparisons of linear mixed model with Tukey test and adjusted p-values with Bonferroni-Holm method. Table presents the fixed effects estimates (Estimates), standard error (Std. Error), *z* statistic and its re**s**pective p-value (Pr (>|*z*|)); alongside to pseudo-R^2^ (R^2^), and the likelihood ratio test chi square statistic (*X*^2^) and its respective p**-**value (Pr(>*X^2^*)). In bold statistically significant t-values (alpha =0.05).

| **Habitat type** | **Estimate** | **Std. Error** | ***z*-value** | **Pr(>\|*z*\|)** | **Pseudo-R^2^** | ***X*^2^** | **Pr(>*X^2^*)** |
| --- | --- | --- | --- | --- | --- | --- | --- |
| Interior-Edge | -15.457 | 7.208 | -2.144 | 0.081 | 0.2141 | 10.43 | ***0.005*** |
| Matrix-Interior | -25.123 | 7.208 | -3.485 | **0.001** |  | | |
| Matrix-Edge | -9.666 | 7.208 | -1.341 | 0.372 |  |  |  |

Table S5. Total and relative of forest fragments with core area 500 m away from the edge in the Brazilian Atlantic Forest. Table shows the range of core area in forest fragments (in ha), total area by range (in ha), standard deviation (SD), number of forest fragments with core areas away of edge effects, and relative number of forest fragments with core areas vs 265,000 fragments in BAF (relative proportion across BAF).

| **Range (ha)** | **Total area**  **(ha)** | **SD** | **Number of core areas** | **relative proportion across BAF** |
| --- | --- | --- | --- | --- |
| **>150** | 3’189,737.2 | 20,724.34 | 806 | 0.304% |
| **(50-150]** | 57,295.3 | 28.38 | 653 | 0.246% |
| **(1 – 50]** | 31,442.7 | 12.94 | 2,259 | 0.852% |
| **<1** | 252 | 0.28 | 868 | 0.328% |
| **TOTAL** | **3’278,727.2** |  | **4,586** | **1.731%** |

**Table S6.** List of the regionally threatened species of vascular epiphytes in our study area in the Brazilian Atlantic Forest. Categories following IUCN parameters. VU= vulnerable; CR; critical; EX: presumed extinct in the wild. Source: Secretaria de Estado do Meio Ambiente, Resolução Sma No 057, 2016.

| **Species name** | **UICN status** | **Family** |
| --- | --- | --- |
| *Tillandsia polystachia* | VU | Bromeliaceae |
| *Rhipsalis crispata* | VU | Cactaceae |
| *Codonanthe carnosa* | VU | Gesneriaceae |
| *Nematanthus crassifolius* | VU | Gesneriaceae |
| *Cirrhaea loddigesii* | CR | Orchidaceae |
| *Cirrhaea longiracemosa* | VU | Orchidaceae |
| *Grandiphyllum divaricatum* | VU | Orchidaceae |
| *Octomeria geraensis* | EX | Orchidaceae |
| *Peperomia quadrifolia* | EX | Peperomiaceae |
| *Peperomia subrubrispica* | EX | Peperomiaceae |

| 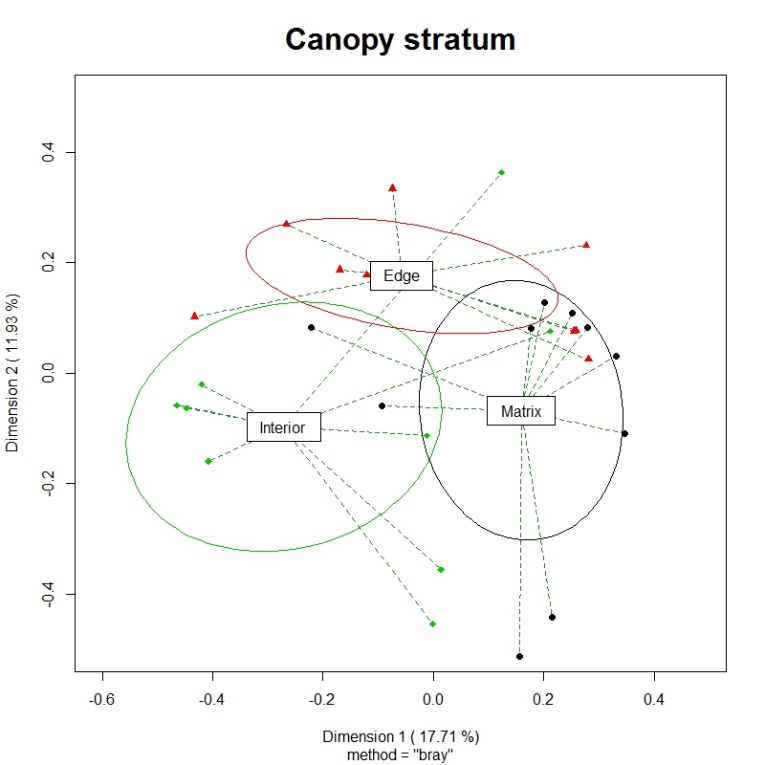  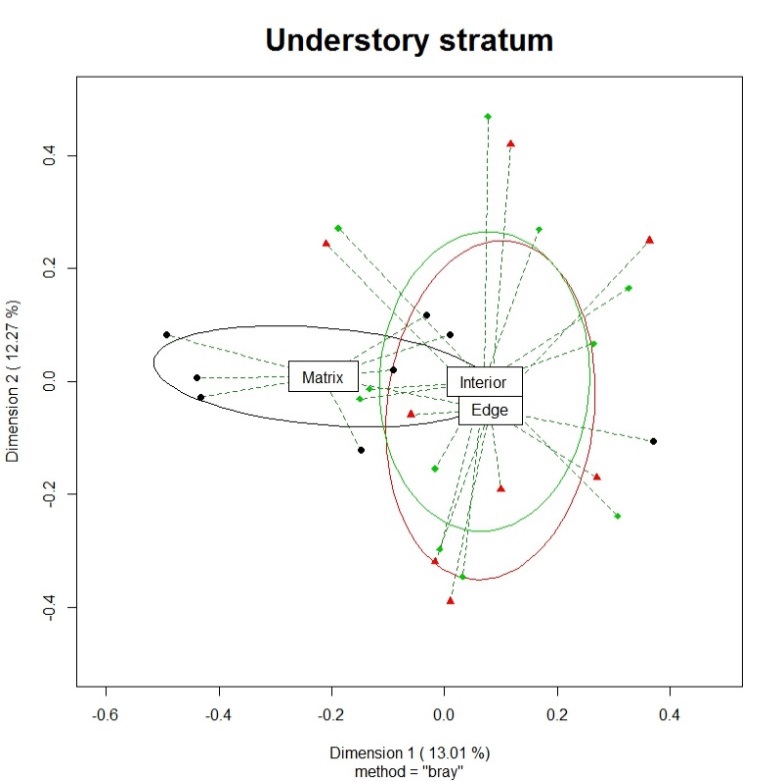  **Figure S1.** Community composition across the interior-edge-matrix gradient of epiphyte vascular plants across canopy (a) and understorey stratum (b), in the Brazilian Atlantic forest. Habitats are depicted by colours, as follows; forest interior (green); forest edge (red); and matrix (black). The permutation test for homogeneity of multivariate dispersion (degrees of freedom= 2, permutations= 9999; F-canopy = 0,270, F-understory = 0,932) in the community composition showed no differences between forest edge and interior (canopy, p= 0.314; understory, p= 0,847), matrix and forest edge (canopy, p= 0,290; understory, p= 0,758), and matrix and forest interior (canopy, p= 0,390; understory, p= 0,823). |
| --- |

**References**

1. Instituto florestal do Estado de São Paulo. Vegetation maps. www.ambiente.sp.gov.br (2010).

2. McGarigal, K., Cushman, S. & Ene, E. FRAGSTATS v4: Spatial Pattern Analysis Program for Categorical and Continuous Maps. (2012).

3. Gustafson, E. J. & Parker, G. R. Using an index of habitat patch proximity for landscape design. *Landsc. Urban Plan.* **29**, 117–130 (1994).

4. Hill, M. O. Diversity and Evenness: A Unifying Notation and Its Consequences. *Ecology* **54**, 427–432 (1973).

5. Chao, A. *et al.* Rarefaction and extrapolation with Hill numbers: A framework for sampling and estimation in species diversity studies. *Ecol. Monogr.* **84**, 45–67 (2014).

6. Hsieh, T. C., Ma, K. H. & Chao, A. iNEXT: an R package for rarefaction and extrapolation of species diversity (Hill numbers). *Methods Ecol. Evol.* **7**, 1451–1456 (2016).

7. Dray, S. & Dufour, A. B. The ade4 package: Implementing the duality diagram for ecologists. *J. Stat. Softw.* **22**, 1–20 (2007).
